# Dentists’, dental students’ and patients’ knowledge, attitudes and practices on artificial intelligence: a scoping review

**DOI:** 10.1186/s12903-026-09081-0

**Published:** 2026-07-01

**Authors:** Georgia Efthymiou, Marianna Charalambous, Elpidoforos S. Soteriades

**Affiliations:** Healthcare Management Program, School of Economics and Management, Open University of Cyprus, 33 Giannou Kranidioti Ave., Latsia, Nicosia, 2220 Cyprus

**Keywords:** Artificial Intelligence, Dentistry, Digital health, KAP study, Diagnostic support, Dental technology, Scoping review

## Abstract

**Introduction:**

Artificial Intelligence (AI) is emerging as a transformative technology in dental practice, promising improved diagnostic accuracy, personalized treatment planning, and workflow optimization.

**Objective:**

We aimed at examining knowledge, attitudes, perceptions, and practices (KAP) associated with the use of Artificial Intelligence in dentistry.

**Materials and methods:**

A comprehensive bibliographic search was conducted following the Preferred Reporting Items for Systematic Reviews and Meta-Analyses extension for Scoping Reviews (PRISMA-ScR) guidelines. Databases included PubMed and Google Scholar, along with official reports from international organizations such as World Health Organization, European Commission, and Organization for Economic Co-operation and Development. Studies published between 2014 and 2025 examining dentists’, dental students’ and patients’ knowledge, attitudes and practices (KAP) related to AI were included. Data extraction included study characteristics, populations, AI applications, and key findings.

**Results:**

Dental professionals generally demonstrate positive attitudes toward AI and recognize its potential benefits. Knowledge levels were generally reported as moderate across studies. However, many studies report limited confidence in using AI-based tools and express concerns regarding data security, ethical implications, and over-reliance on automated systems. The need for structured training and education in AI applications was consistently highlighted.

**Conclusion:**

This scoping review indicates that dentists and dental students demonstrate moderate knowledge, generally positive attitudes, and limited clinical practices regarding Artificial Intelligence. The findings reveal a clear gap between awareness and implementation, primarily driven by insufficient training and practical exposure. Strengthening education and addressing barriers to adoption are essential to support the effective integration of AI in dental practice.

## Background

The integration of digital technologies in healthcare depends not only on technological advancement but also on dental professional’s knowledge, attitudes, and practices. Artificial Intelligence (AI) has emerged as a transformative tool in dentistry, enhancing diagnostic accuracy, treatment planning, and evidence-based decision-making across specialties such as radiology, orthodontics, endodontics, periodontology, prosthodontics, implantology, and surgical robotics [1, 2]. Algorithms such as Support Vector Machines, Random Forests, and Convolutional Neural Networks enable automated risk assessment and precise applications, including skeletal maturation analysis, computer-aided design / computer-aided manufacturing (CAD/CAM) prosthetic design, implant placement, caries detection, periodontal disease classification, and identification of cysts, tumors, and metastatic lymph nodes, often matching or exceeding clinician accuracy [1, 3].

Despite these capabilities, the level of awareness, attitudes, and readiness for AI adoption among dentists and dental students’ remains insufficiently explored and the available evidence is limited and fragmented. Concerns regarding algorithm reliability, data privacy, ethical considerations, legal responsibility, and clinical autonomy may further influence its acceptance and integration into dental practice [2, 4]. Barriers also include insufficient training, lack of standardized guidelines, and limited curricular integration. AI is generally seen as supportive for enhancing efficiency, reducing errors, and strengthening preventive and diagnostic care rather than replacing dentists [1, 5].

AI’s rapid expansion introduces ethical and practical challenges, including algorithm transparency, dataset bias, patient data protection, and accountability. Effective implementation requires collaboration among dental professionals, data scientists, engineers, and health-informatics experts to ensure accurate interpretation, workflow integration, and ethical compliance [6, 7]. Continuous education, structured training, and clear regulatory frameworks are essential.

This scoping review synthesizes current evidence on dentists’, dental students’ and patients’ knowledge, attitudes, perceptions, and behaviors toward the use of AI, and identifies adoption factors, providing guidance for responsible AI implementation, professional education and policy, promoting precise, efficient, and equitable dental care.

## Methodology

**Fig. 1:**
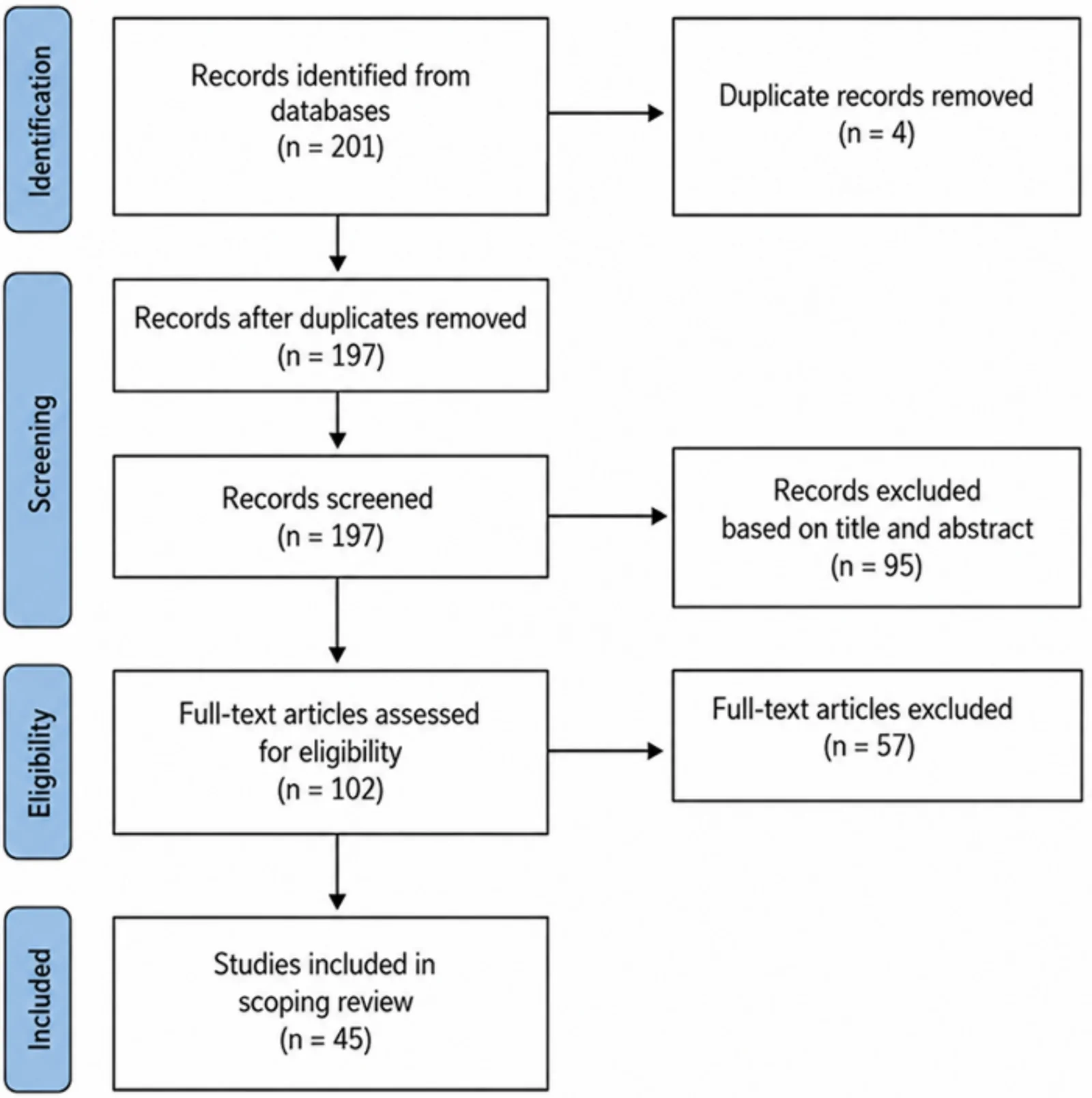
Prisma Flowchart of the study selection process

Our study aimed at systematically mapping and synthesizing the existing evidence on dentists’, dental students’ and patients’ knowledge, attitudes, perceptions, and practices (KAP) regarding the use of Artificial Intelligence (AI) in dental practice and education. The review was conducted in accordance with the Preferred Reporting Items for Systematic Reviews and Meta-Analyses extension for Scoping Reviews (PRISMA-ScR) guidelines including the number of records identified, duplicates removed, records screened, full-text articles assessed for eligibility, excluded studies with reasons, and final included studies (Fig. 1). A scoping review design was chosen for this study due to the exploratory nature of the research question and the aim to map the breadth of existing literature on knowledge, attitudes, perceptions, and practices regarding Artificial Intelligence in the field of dentistry. Unlike systematic reviews, which focus on narrowly defined clinical questions and typically involve risk of bias assessment and quantitative synthesis, scoping reviews are better suited for emerging and rapidly evolving fields. Given the heterogeneity of study designs, populations, and outcomes, as well as the fast-paced development of Artificial Intelligence technologies and applications in dentistry, a scoping review methodology was considered most appropriate to identify key concepts, summarize existing evidence, and highlight knowledge gaps without restricting inclusion based on methodological uniformity. As a scoping review based exclusively on previously published literature, ethical approval was not required.

A comprehensive bibliographic search was performed between January 2014 and December 2025 using the electronic databases of PubMed and Google Scholar. In addition, official reports and policy documents from international organizations, including the World Health Organization (WHO), the European Commission, and the Organization for Economic Co-operation and Development (OECD), were reviewed when they specifically addressed Artificial Intelligence in healthcare or dentistry. PubMed was used as the primary biomedical database due to its extensive coverage of dental and medical literature, while Google Scholar was used as a supplementary source to enhance search sensitivity and capture additional relevant and grey literature. Given the scoping nature of this review, this search strategy was considered appropriate to map the breadth of available evidence rather than to perform an exhaustive systematic search across multiple indexed databases.

The search strategy employed a combination of Medical Subject Headings (MeSH) terms and free-text keywords related to Artificial Intelligence and dentistry. Key search terms included: *“Artificial Intelligence in Dentistry”*, *“Dentists’ Knowledge Attitudes and Practices”*, *“AI in Dental Practice”*, *“Machine Learning in Dentistry”*, *“Deep Learning in Dentistry”*, and *“Dental Education and Artificial Intelligence”*. A pilot search was initially conducted in PubMed to refine the search strategy and identify relevant keywords and indexing terms. Based on this preliminary search, the final search strategy was developed to maximize sensitivity and specificity. Boolean operators (AND/OR) were used across all databases to refine the search strategy. The final Boolean search strings in PubMed were (“Artificial Intelligence” OR “Machine Learning” OR “Deep Learning”) AND (“Dentistry” OR “Dental Practice” OR “Dental Students”) AND (“Knowledge” OR “Attitudes” OR “Practices” OR “Perceptions”). Filters applied included publication date (2014–2025), language (English or Greek), and human studies where applicable. Google Scholar search used a simplified keyword-based search strategy due to platform limitations. The search terms included “Artificial Intelligence” AND dentistry AND (knowledge OR attitudes OR practices OR perception). Results were screened based on relevance, and the first eligible records sorted by relevance were reviewed. No advanced field restrictions or indexing filters were available in this database.

The literature selection followed a three-stage screening process: (1) title and abstract screening to assess relevance, (2) full-text review of potentially eligible studies, and (3) final inclusion based on predefined eligibility criteria. Two independent reviewers (MC and ESS), with academic and research background in healthcare management and oral health sciences, conducted the screening process. In addition, a dentist (GE) with clinical training in dentistry participated in the review process to ensure subject-matter expertise. Any disagreements were resolved through discussion and consensus. Only peer-reviewed articles and official reports published between January 2014 and December 2025 in English or Greek were considered. The year 2014 was selected as the starting point of the search period as it marks the beginning of the modern deep learning era, following major advancements in convolutional neural networks and the widespread application of artificial intelligence techniques in medical imaging and healthcare. From this period onwards, AI research in dentistry began to expand significantly, particularly in image-based diagnostics and clinical decision-support systems. Therefore, limiting the search to studies published from 2014 ensured the inclusion of contemporary, clinically relevant, and methodologically comparable evidence reflecting current AI applications in dentistry.

### Inclusion criteria

Studies were eligible if they explicitly investigated Artificial Intelligence (AI) in dentistry, encompassing applications such as machine learning algorithms, deep learning models, neural networks, computer vision systems, natural language processing approaches, and AI-driven decision-support systems used in either dental practice or education. Eligible populations included dentists, dental students, specialists, other dental professionals, and patients, provided that the studies assessed at least one relevant outcome related to knowledge, attitudes, perceptions, behaviors, or practices regarding the use of AI in dentistry. A broad range of methodological designs was considered, including quantitative, qualitative, and mixed-methods studies, as well as cross-sectional surveys, observational research, interview-based studies, systematic reviews, and scoping reviews. Data extraction was undertaken using a pre-designed chart that captured key study characteristics, including author, year of publication, country, study design, and main findings. Within this process, operational definitions were consistently applied, whereby knowledge referred to factual awareness or understanding of AI concepts and applications in dentistry, attitudes encompassed beliefs, perceptions, and overall evaluations of AI, practices and behaviours reflected the actual use or implementation of AI technologies in clinical or educational contexts, and perceptions described subjective interpretations or opinions concerning the utility, risks, and feasibility of AI.

### Exclusion criteria

Studies were excluded when they focused on digital dentistry, teledentistry, or general digital health without AI being a central component of the intervention or outcome. Research limited exclusively to the technical development, validation or performance evaluation of AI algorithms was also excluded when it did not address dentists’, dental students’ or other dental professionals’ knowledge, attitudes, perceptions, or practices. In addition, non-primary publications such as editorials, commentaries, letters and conference abstracts without full-text availability were excluded. In addition, theses and dissertations, non-English or non-Greek language publications, studies with inaccessible full texts, as well as studies conducted outside the context of dental or oral healthcare were excluded.

## Results

A total of 201 records were identified through database searching. After removing 4 duplicates, 197 records were screened based on title and abstract. Of these, 95 records were excluded as they did not meet the inclusion criteria. Subsequently, 102 full-text articles were assessed for eligibility. Following full-text review, 57 articles were excluded for reasons such as lack of relevance to Artificial Intelligence in dentistry, absence of knowledge, attitudes, practices (KAP) outcomes, or focus on purely technical AI development without clinical or behavioral assessment. Finally, 45 studies were included in the scoping review (Table 1).

**Table 1:** CharacteristicsSummary of Selected Studies For Scoping Review

A.A.	Authors, Year	Place	Study type	Target population	Key Features
1	Pringle et al. 2024 [9]	South Tamil Nadu in India	Cross sectional online Survey	Dentists and non-dentists population	Survey of dentists and non-dentists. Evaluates perception and attitude on AI in dentistry for diagnosis and treatment planning.
2	Jeong et al. 2024 [19]	South Korea	Online survey	Dental students and Dentists	Explores differences in AI perceptions between dental students and dentists based on seniority.
3	Dashti et al. 2024 [10]	Iran	A systematic Review	Dentists and dental students	Systematic review assessing knowledge, attitudes, and perspectives on AI in dentistry. Highlights gaps in understanding.
4	Barnawi et al. 2025 [17]	Saudi Arabia	Cross-sectional	Dentists and dental/oral health practitioners	Assessing dentists’ knowledge, attitudes, and use of AI and robotics in the care of persons with disabilities.
5	Kostov et al. (2025) [32]	Bulgaria	Cross-Sectional	Dentists, dental students, and patients	Study exploring dentists’ preferences regarding AI integration in clinical practice. Findings show strong preference for hybrid models combining AI-assisted data analysis with clinician-led decision-making.
6	Mohammed et al. 2025 [31]	Sudan	Cross-sectional	Dental students	Questionnaire-based study among dental students evaluating AI knowledge sources, level of training, and attitudes toward AI in dentistry.
7	Zia et al. 2024 [20]	Pakistan	Cross-sectional survey	Dental students, graduates, postgraduates, and professionals	Cross-sectional survey examining dentists’ perceptions of AI and robotics in dentistry. Most participants perceived AI as beneficial for improving treatment quality, diagnostic accuracy, and surgical precision.
8	Hall et al. 2023 [14]	Egypt	Online based Cross sectional Study	Egyptian Dentists (dentists with different levels of experience, specialties, and working in different health sectors)	Cross-sectional study evaluating Egyptian dentists’ knowledge, awareness, and perception toward digital dentistry.
9	Eschert el.al. 2022 [4]	Germany	Online Survey	Random sample of 1500 dentists, specialist dentists and oral and craniomaxillofacial surgeons randomly chosen from the membership list of the Dental Association of Westfalen-Lippe, Germany.	Survey on clinicians attitudes, knowledge, and expectations regarding AI in dentistry and maxillofacial surgery.
10	Usha et al. 2025 [26]	India	Cross-sectional	Undergraduate and postgraduate students	Cross-sectional survey assessing dentists’ knowledge, attitudes, and practices regarding Artificial Intelligence in dentistry.
11	Bedia et al. 2024 [29]	Spain	Descriptive online questionnaire-based survey	Dental residents and dentists	Descriptive cross-sectional survey conducted among dentists and dental residents in the Navi Mumbai region. Findings indicate generally positive attitudes toward AI, particularly for diagnostic accuracy, treatment planning, and workflow efficiency.
12	Katkade et al. 2024 [18]	Maharashtra state	Questionnaire survey Cross sectional study	400 Dental professionals	Evaluates perceptions and attitudes of dental professionals toward digital dentistry via questionnaire.
13	Singh et al. (2023) [2]	India	Questionnaire-based cross-sectional study online survey	Dentists and dental students	Assesses attitudes, perceptions, and barriers of dental professionals toward AI in India among students and dentists.
14	Kalaimani et al. (2023) [1]	India	Questionnaire-based cross-sectional online survey	Dentists and dental students	Cross-sectional survey measuring knowledge, attitude, and practice (KAP) of AI among dentists and dental students.
15	Ahmed et al. 2021 [40]	Not clearly specified	Systematic review	Patient/simulator faciodental images, radiographs, CAD/CAM, Virtual dental models	Reviewed AI techniques applied in dentistry, including machine learning, deep learning, and neural networks across diagnostics, radiographic interpretation, treatment planning, and outcome prediction. Reported that AI models generally show high performance in imaging-based tasks (e.g., caries detection, periodontal and radiographic analysis), but emphasized limitations such as small datasets, lack of external validation, heterogeneity of methods, and need for clinical translation and standardization.
16	Karan-Romero et al. 2023 [30]	Mexico	Observational, descriptive, and cross-sectional	Dental students	Cross-sectional survey assessing students’ perceptions of AI applications in dentistry. Results showed strong acceptance of AI for radiographic diagnosis, disease detection, treatment planning, implant planning, and quality control
17	Lin et al. (2023) [22]	Malaysia	One-to-one in-depth online interviews	Dental technicians	Qualitative interviews exploring dental technicians’ perceptions of AI integration in laboratory practice.
18	Karnik et al. 2024 [45]	India	Narrative review.	Prosthodontists, implantologists and dental researchers	Reviewed applications of robotics and artificial intelligence in prosthodontics and implantology, including implant planning, guided surgery, prosthesis design, and workflow automation. Highlighted benefits such as improved precision, reduced human error, and enhanced treatment outcomes, while also discussing limitations like high cost, technical complexity, limited clinical validation, and the need for clinician training and regulatory frameworks.
19	Murali et al. 2023 [37]	India	Cross-sectional questionnaire-based study	Dental students and professionals	Questionnaire-based cross-sectional study evaluating attitudes toward AI integration in dentistry. Findings indicate general readiness to adopt AI for analytical and diagnostic tasks, while hesitation remained regarding direct clinical implementation.
20	Vashisht et al. 2024 [8]	India	Scoping Review	Electronic databases were searched for scientific research articles	There are various Artificial Intelligence models being used in various fields of dentistry with high accuracy, sensitivity and specificity.
21	Fritsch et al. 2022 [3]	Germani	Cross-sectional study	Patients and their companions at a tertiary referral hospital	Assessed perceptions, attitudes, technical affinity, and acceptance of AI in healthcare using a paper-based questionnaire. Most participants had positive attitudes toward AI, but preferred physician supervision and human responsibility in diagnosis and treatment decisions. Older age, female gender, lower education, and lower technical affinity were associated with greater caution toward AI use.
22	AL- Medfa MK, et al. 2023 [5]	Bahrain	Cross-sectional study	Physicians practicing in Bahrain	Investigated physicians’ knowledge and attitudes toward artificial intelligence in medicine; explored perceived benefits (improved diagnostics, efficiency, and decision-making) and drawbacks (ethical concerns, job replacement, privacy, lack of training); found generally positive attitudes toward AI but identified need for education and training in AI applications in healthcare.
23	Bonny et al. 2023 [6]	United Arab Emirates	Narrative review/literature review	Dental professionals, researchers and dental health care systems	Reviewed contemporary applications of artificial intelligence in dentistry including diagnosis, radiographic interpretation, orthodontics, prosthodontics, endodontics, and treatment planning. Highlighted benefits such as improved diagnostic accuracy and efficiency, discussed challenges including ethical issues, data privacy, implementation costs, and need for professional training.
24	Patrzyk et al. 2022 [7]	Poland	Cross- sectional survey study	Polish dermatologists and dermatology trainees	Explored attitudes toward modern technologies and artificial intelligence in medicine among dermatologists and trainees. Assessed knowledge, acceptance, and concerns regarding AI use in dermatology. Participants recognized potential benefits in diagnostics and clinical support but expressed concerns about reliability, ethical issues, and replacement of physicians.
25	Sur et al. 2020 [11]	India	Cross-sectional study	Oral radiologists, dental professionals and postgraduate dental students in India	Assessed knowledge, attitudes, and perceptions regarding the future role of artificial intelligence in oral radiology. Participants generally showed positive attitudes toward AI applications in diagnosis and imaging interpretation, highlighted concerns about lack of training, dependence on technology, and possible reduction in professional roles.
26	Liehner et al. 2023 [13]	Germany	Cross-sectional survey study	General public/adult population	Examined public perceptions, attitudes, and trust toward artificial intelligence, evaluated acceptance of AI technologies across different domains. Findings showed mixed attitudes, with recognition of AI benefits such as efficiency and innovation alongside concerns regarding privacy, transparency, ethics, and loss of human control. Trust in AI varied according to familiarity and perceived risk.
27	Razdan et al. 2025 [21]	India	Online Cross-sectional questionnaire study	Dentists involved in pediatric dentistry	Examined public perceptions, attitudes, and trust toward artificial intelligence. Evaluated acceptance of AI technologies across different domains. Findings showed mixed attitudes, with recognition of AI benefits such as efficiency and innovation alongside concerns regarding privacy, transparency, ethics, and loss of human control; trust in AI varied according to familiarity and perceived risk. Investigated dentists’ knowledge, perceptions, and attitudes regarding artificial intelligence in pediatric dentistry. Participants generally demonstrated positive perceptions toward AI applications in diagnosis, treatment planning, and patient management. Identified limited formal training and concerns about ethical issues, accuracy, and dependence on technology as barriers to implementation.
28	Kukreja et al. 2025 [12]	India	Cross-sectional questionnaire study	Dental undergraduate students	Assessed knowledge and awareness of machine learning and artificial intelligence among dental undergraduate students, evaluated familiarity with AI applications in dentistry and education. Findings suggested moderate awareness with generally positive attitudes toward integrating AI into dental practice and training, while highlighting the need for improved education and formal curriculum inclusion.
29	Elchaghaby et al. 2025 [15]	Egypt	Cross-sectional survey study	Egyptian dental students	Investigated dental students’ knowledge, attitudes, and perceptions toward artificial intelligence in dentistry. Participants generally demonstrated positive attitudes toward AI integration in dental education and clinical practice; identified gaps in formal AI knowledge and emphasized the need for curriculum development, training, and ethical guidance regarding AI applications in dentistry.
30	Thakkar et al. 2025 [16]	India	Cross-sectional questionnaire study	Dentist practicing in Udaipur	Evaluated awareness of artificial intelligence in oral medicine and radiology among dentists. Assessed understanding of AI applications in diagnosis and radiographic interpretation. Findings indicated growing awareness and positive attitudes toward AI adoption, while emphasizing the need for additional training and education to support implementation in dental practice.
31	Hamd et al. 2023 [23]	United Arab Emirates	Cross-sectional study	Dentists and dental professionals ( including trainees, depending on sample)	Examined knowledge, perceptions, and future prospects of artificial intelligence integration in dentistry. Assessed awareness of AI applications in clinical practice and diagnostics. Findings indicated generally positive attitudes toward AI adoption, with recognition of its potential to improve efficiency and diagnostic accuracy, alongside concerns about insufficient training, ethical considerations, and implementation barriers in dental settings.
32	Ghasemian et al. 2025 [24]	Iran	Cross-sectional survey study	Dental students	Assessed dental students’ attitudes toward artificial intelligence in dentistry, including perceived usefulness, readiness to adopt AI tools, and concerns. Findings indicated generally positive attitudes toward AI integration in dental education and clinical practice, with highlighted barriers such as limited training, lack of curricular exposure, and ethical considerations.
33	Mishra et al., 2025 [25]	India	Cross-sectional study questionnaire	Dentists and dental professionals(including students in some samples)	Assessed knowledge, attitudes, and perceptions regarding the use of robotics and artificial intelligence in dentistry. Explored awareness of emerging digital technologies in clinical practice; findings generally showed positive attitudes toward adoption of AI and robotics for diagnosis and treatment support, while highlighting barriers such as limited training, cost, lack of curriculum integration, and ethical and implementation concerns.
34	Ivanisevic et al. 2024 [27]	Croatia	Cross-sectional study	Dentists practicing in Croatia	Assessed knowledge, attitudes, and perceived barriers regarding artificial intelligence in dentistry. Evaluated readiness for AI adoption in clinical practice. Findings showed generally positive attitudes toward AI and recognition of its potential benefits in diagnostics and treatment planning, while identifying key barriers such as insufficient training, limited exposure in education, cost concerns, and ethical and implementation challenges.
35	Hegde et al. 2024 [28]	Australia	Cross-sectional study	Dentists practicing in Australia	Examined dentists’ attitudes toward artificial intelligence in dentistry, including perceived usefulness, acceptance, and readiness for adoption; identified generally positive attitudes toward AI integration in clinical practice, particularly for diagnostic support and workflow efficiency. Also highlighted concerns about training needs, ethical implications, data privacy, and reliability of AI systems.
36	Elburki et al. 2025 [33]	Libya	Cross-sectional study	Dentists	Assessed dentists’ knowledge, perceptions, and attitudes toward artificial intelligence in dentistry. Evaluated awareness and readiness for AI adoption in clinical practice. Findings generally indicated moderate knowledge with positive attitudes toward AI, alongside concerns about lack of training, limited exposure, and ethical/implementation challenges.
37	Alhazmi et al. 2022 [34]	Saudi Arabia	Cross-sectional survey study	Dentists	Evaluated dentists’ knowledge and clinical practices regarding artificial intelligence; assessed awareness of AI applications in dental diagnosis and treatment. Findings suggested limited to moderate knowledge but generally positive attitudes toward AI adoption, with barriers including lack of training, limited exposure, and concerns about reliability and implementation in routine dental practice.
38	Kahveci et al. 2024 [35]	Turkey	Cross-sectional survey	Dental students	Evaluated dental students’ attitudes and perceptions toward AI in dentistry using a face-to-face questionnaire among 72 students. Most participants reported basic AI knowledge (61%) and awareness of AI applications in dentistry (75%). The majority believed AI would improve professional success and advance dentistry, while fewer believed AI would replace dentists. The study recommended integrating AI education into undergraduate and postgraduate dental curricula.
39	Alshahrani et al. 2025 [36]	Saudi Arabia	Cross-sectional survey	Dental students and interns	Assessed knowledge and perceptions of AI in dental education using a structured questionnaire. Participants showed moderate knowledge and generally positive perceptions toward AI integration in dentistry. Many expressed interest in formal AI training and inclusion in the curriculum. Concerns were reported regarding ethical issues, data privacy, and the potential impact on clinical decision-making.
40	Ezzeldin et al. 2025 [38]	Egypt	Cross-sectional study	Dental students	Assessed knowledge and attitudes toward AI and robotics in dentistry using a structured questionnaire. Findings indicated limited to moderate knowledge but generally positive attitudes toward AI applications. Most students expressed interest in learning more about AI and supported its integration into dental education. Concerns were noted regarding job replacement, ethical implications, and lack of formal training.
41	Bahadir et al. 2025 [41]	Turkey	Cross-sectional survey	Dental patients	Evaluated patients’ attitudes and acceptance of artificial intelligence in dentistry using a structured questionnaire. Overall, participants generally showed positive or neutral attitudes toward AI-assisted dental care, with varying levels of trust depending on application type. Higher acceptance was typically associated with younger age, higher education level, and prior awareness of AI. Concerns included data privacy, lack of human interaction, and uncertainty about diagnostic reliability.
42	Prabhu et al. 2024 [39]	India	Narrative review	Dental professionals, researches and students	Reviewed the current and future applications of artificial intelligence in dentistry, including diagnosis, treatment planning, radiographic interpretation, predictive analytics, and clinical decision support. The article highlights AI’s potential to improve diagnostic accuracy and efficiency while emphasizing challenges such as ethical concerns, data privacy, lack of standardized datasets, and the need for clinician training and regulatory frameworks.
43	Schwendicke et al. 2020 [42]	Germany	Narrative review	Dental researchers, clinicians and academic community	Discussed the opportunities and challenges of artificial intelligence in dentistry, including applications in radiology, diagnostics, treatment planning, and risk prediction. The article emphasizes AI’s potential to enhance diagnostic accuracy and workflow efficiency, while highlighting key challenges such as data quality, validation, ethical/legal concerns, explainability (“black box” problem), and the need for robust clinical integration and regulation.
44	European Commission 2012 [43]	Brussels, Belgium	Policy document/eHealth Action plan	European Union health care systems, policy makers, health care providers and digital health stakeholders	Outlined the EU strategic framework for eHealth (2012–2020), aiming to promote digital health adoption, interoperability of health systems, patient-centered care, and cross-border health data exchange. Emphasized ICT integration in healthcare, improved efficiency, quality of care, and support for innovation in digital health services across EU member states.
45	Organization for Economic Co-operation and Development 2020 [44]	Paris, France	Policy report	OECD member countries, health policy makers, health system planers and public health authorities	Examines how health data and digital technologies can strengthen health systems, improve decision-making, and enhance care quality. Highlights the importance of data governance, interoperability, digital transformation, and responsible use of emerging technologies (including AI) to support efficiency, patient outcomes, and health system resilience across OECD countries.

The literature indicates that dentists generally have positive attitudes toward Artificial Intelligence (AI), recognizing its potential to enhance diagnostic accuracy, treatment planning, and clinical efficiency. These perceptions are closely associated with AI applications that are already implemented or rapidly emerging across dental specialties [8].

### Perceived roles of artificial intelligence in dental practice

AI applications in oral and maxillofacial radiology are among the most widely accepted, as reported in the included studies, with machine learning and deep learning algorithms being increasingly used in panoramic radiography, cone-beam computed tomography (CBCT), and intraoral imaging for the detection of caries, periapical pathology, periodontal bone loss, fractures and anatomical landmarks. The perceived high accuracy and reproducibility of these systems contribute to clinicians’ trust and willingness to integrate AI into practice [8].

In orthodontics and prosthodontics, AI is perceived as supporting automated cephalometric landmark identification, facial analysis, digital treatment planning and computer-aided design of prosthetic restorations and implants. These tools are mainly perceived as decision-support systems that reduce treatment time and human error rather than replacing dentists [8]. In restorative dentistry, endodontics, and periodontology, AI contributes to caries detection, root canal anatomy identification, periodontal assessment, and outcome prediction. However, dentists express greater caution due to concerns regarding algorithm transparency, population generalizability, and medico-legal responsibility [8].

Beyond clinical practice, AI is increasingly applied in dental education and practice management, including virtual simulations, digital learning platforms, automated assessment systems, appointment scheduling, and patient data management. These applications are generally perceived as less controversial and often represent dentists’ initial exposure to AI technologies [8]. Despite growing acceptance, a persistent gap remains between awareness of AI applications and understanding of their principles and ethical implications, underscoring the need for structured undergraduate and postgraduate AI education to support responsible and effective integration into dental practice [8].

### Dentists’ knowledge of AI

Dental students demonstrated knowledge levels ranging from approximately 48% to 58.6%. Evidence indicates that dentists’ knowledge of Artificial Intelligence (AI) remains moderate and heterogeneous across populations. A report from South Tamil Nadu, India, described that 40.5% of participants were unaware of AI applications in caries detection, while 43.9% recognized its use in radiographic interpretation and 42.4% in orthodontic treatment planning [9]. A total of 44.7% of participants believed that clinicians’ expertise exceeds AI-based diagnostic performance. Dentists demonstrated higher awareness and acceptance of AI than non-dentists, with 44.3% of dentists versus 25.5% of non-dentists being aware of robotic devices, and 57% supporting AI’s potential to improve treatment quality. In general, 66.7% reported moderate knowledge of AI [9]. 

Similarly, other studies found that dental students and practicing dentists have moderate AI knowledge (dentists: 71.8%, students: 58.6%) and are optimistic about its clinical potential, though this knowledge is not consistently applied in practice [10, 11]. Among students, awareness varies. Findings show that 67% had heard of AI and 35% were familiar with machine learning, with clinical students demonstrating higher knowledge than preclinical peers [12]. Further studies concluded that overall, AI awareness in dentistry remains unsatisfactory [1]. While most participants recognize AI’s benefits, only 62.8% are familiar with its basic principles, highlighting strong student interest in AI education at undergraduate and postgraduate levels [2]. Despite widespread exposure to AI technologies, a lack of understanding of their underlying mechanisms was reported, which may lead to overestimation or underestimation of AI capabilities [13].

Institutional and demographic factors were also reported to influence AI knowledge. Studies in Egypt indicated higher awareness among dentists in academic settings, prosthodontists, and urban practitioners, while rural dentists and those in governmental clinics exhibited moderate knowledge but high perception of digital dentistry, emphasizing the need for structured education and policy support [14]. Egyptian students were shown to have 48–49% basic knowledge [15, 16]. A study similarly noted that dentists generally possess basic AI awareness but lack understanding of development, validation, and ethical considerations, with higher knowledge among younger dentists, specialists, and postgraduate students [8].

More limited knowledge was reported in some populations, with findings indicating that only 32% of dentists were familiar with AI concepts, highlighting insufficient education as a barrier to integration, especially in care for people with disabilities [17].

### Attitudes and perceptions of dentists on AI

Evidence indicates that dentists generally hold positive attitudes toward Artificial Intelligence (AI), though perceptions vary with knowledge, exposure, and context. Studies report that most dental professionals and students view AI as complementary rather than a replacement for clinicians, recognizing its potential in diagnosis, treatment planning, surgical procedures, implant placement, tooth alignment, and education [8–11, 17−22].

Findings show that people prefer human judgment when it conflicts with AI [9], recognize AI’s benefits for productivity and education [20], and expect AI to be fully adopted in practice within 8–11 years [22]. Knowledge level and prior AI exposure strongly influence attitudes, with greater familiarity correlating with trust and optimism [4, 13].

Persistent concerns include privacy, ethical and legal liability, false positives, transparency, and regulatory requirements [2, 8, 11, 12, 15, 16, 19, 23, 24]. Structured training, curriculum integration, and interdisciplinary collaboration with AI engineers are recommended to enhance understanding, mitigate fear, and support safe implementation. In general, attitudes toward AI in dentistry are largely positive, yet actual adoption is limited.

### Dentists’ behaviors and practices of AI

Literature shows that integration of Artificial Intelligence (AI) in dentistry influences student education, workflow, diagnosis, and treatment. Although AI use and benefits are increasingly recognized, clinical use remains limited [8, 12, 15]. Furthermore a study reported insufficient practical experience with CAD/CAM technologies, recommending incorporation of digital dentistry training at undergraduate and postgraduate levels [18].

Although 75% recognized AI’s potential, only 40% felt confident using it without training, and 55% expressed job replacement concerns. Several studies reported 88% awareness among Indian students and professionals, with 81% understanding AI functions [15, 16].

In clinical practice, AI adoption is variable. Some dentists use AI for radiographic interpretation, treatment planning, and practice management, but overall experience is limited. AI is primarily assistive and integrated with clinicians [8, 23]. In orthodontics, 68.8% believed AI could replace manual methods, 87.5% noted reduced data analysis time, and 72.7% considered results more accurate, with knowledge mainly acquired through lectures and training [22].

Internationally, 65% of dentists report low or no AI usage, and only 6.3% have excellent knowledge [23]. Lin et al. found 35% had applied AI clinically, with 62% believing some functions could be delegated to AI [22]. Barnawi et al. reported 59.2% of dentists working with persons with disabilities used AI for at least one task, with prior training as the strongest predictor [17]. High awareness among students and professionals was also observed, particularly for radiograph analysis, periodontal disease detection, and CAD/CAM processes, although skepticism about replacement remains prevalent [24, 25].

Dentists’ willingness to adopt AI is shaped by perceived benefits and training opportunities. Zia et al. found that 55.2% recommended AI for treatment, 51% were willing to receive AI-assisted care and 73.4% desired future training [20]. Usha et al. highlighted strong interest in proper training and institutional support [26]. Digital tool adoption is rising as 64% use digital impressions and 87% digital radiography, with readiness for diagnostic tasks despite primarily conceptual experience [27, 28] and similarly 65% are likely to incorporate AI within five years [29].

Strong acceptance for clinical and educational AI integration has been observed [30, 31], with preference for hybrid models combining AI and clinicians’ decisions [32]. Limited adoption persists due to lack of training, access, ease of use, infrastructure, cost, patient acceptance on education and institutional support [33, 34], while students remain open to clinical AI but uncertain about curriculum inclusion [35].

### Factors affecting dentists’ use of AI

The adoption and integration of Artificial Intelligence (AI) in dental practice are influenced by multiple factors, including age, gender, professional experience, specialty, work environment, access to technology, resources and institutional support. Younger dentists, postgraduate students, and those in academic, research-oriented, or private settings are generally more familiar with AI and more ready to implement it than older practitioners or those in public clinics. Prior exposure to digital dentistry, robotics, or AI-related research further increases adoption likelihood [8, 22, 24, 25]. Female students are more aware of ethical, privacy, and safety concerns, while male students and specialists report greater confidence in applying AI to diagnostics and treatment planning. Younger professionals also exhibit more optimism and are more likely to integrate AI in education or clinical practice, reflecting generational differences in technology acceptance [10, 24, 28, 31].

Education and formal training critically determine knowledge and implementation. Insufficient undergraduate or postgraduate AI education, limited workshops, and lack of continuing professional development restrict effective use. Prior training in AI was the only independent predictor of adoption among dentists working with people with disabilities, while readiness correlated with involvement in clinical or research projects [10, 14, 17, 22, 36, 37].

Dentists in well-equipped academic or private institutions are more likely to use AI for radiographic interpretation, treatment planning, and simulations, whereas those in rural or under-resourced settings face infrastructural barriers. Practical adoption is further limited by cost, regulatory uncertainty, complexity, and liability or patient safety concerns [ 9, 14, 26, 29, 36].

Finally, the type of AI applications and context of use affect adoption. Dentists generally prefer hybrid models where AI supports but do not replace clinicians. Positive perceptions of AI, is potential for efficiency, accuracy, and decision-making alone is insufficient. Structured training, institutional readiness, and experiential opportunities are essential to translate favorable attitudes into clinical practice [20, 22, 32, 33, 35, 37].

## Discussion

The literature consistently highlights the significant potential of Artificial Intelligence (AI) to enhance diagnostic accuracy, improve imaging interpretation, support personalized treatment planning, and reduce human error in dentistry. Dental professionals and students demonstrate moderate to high awareness of AI applications, particularly in radiographic analysis, cephalometric evaluation, lesion detection, CAD/CAM procedures, implant planning, and treatment planning. The transformative role and emerging clinical utility of AI in dentistry have been widely emphasized in recent studies [12, 22, 38–40]. In practice, AI is predominantly applied in analytical, diagnostic, and treatment planning tasks and is rarely used autonomously, reflecting a preference for hybrid models in which AI supports rather than replaces clinical decision-making [17, 32, 37, 41]. This dual nature of AI implementation has been described as both an opportunity and a challenge, depending on clinical context and technological maturity. Regardless of the generally positive attitudes, persistent concerns include ethical and data privacy issues, lack of transparency in AI decision-making, liability, and potential professional replacement, reinforcing the perception of AI as a complementary rather than substitutive tool [4, 10, 34, 42]. A recurring finding across the literature is the insufficient level of formal education and hands-on training in AI, which limits professional confidence and hinders its effective integration into clinical practice. In this context, prior exposure to AI through lectures, workshops, clinical projects, and structured training programs has been reported as an important determinant of adoption [22, 35, 36]. Institutional support, technology access, and infrastructure availability further affect uptake, with higher usage in academic or private settings compared to resource-limited clinics [14, 29]. Generational differences are also evident, as students and younger professionals tend to demonstrate greater optimism and readiness for AI adoption, whereas practicing dentists favor more cautious, supervised use while maintaining clinical autonomy [28, 31].

A critical challenge in the implementation of AI in dentistry is the presence of bias in training datasets. Many AI models are developed using limited, non-representative, or geographically restricted datasets, which may compromise their generalizability across diverse populations and clinical settings. This limitation can lead to variability in diagnostic accuracy and unequal performance across patient groups. Furthermore, awareness of such biases among dental professionals appears to be limited, potentially influencing trust and adoption. These findings align with the variability in knowledge and confidence levels reported across studies included in this review. Therefore, validation using heterogeneous and representative datasets is essential to ensure fairness, reliability, and safe clinical application of AI technologies [40].

In addition to global evidence, this scoping review provides a regionally relevant synthesis with particular applicability to Cyprus and the broader European context, where evidence on AI adoption in dentistry remains limited. By integrating findings from diverse international settings, this study bridges the gap between global technological advancements and their implementation in smaller healthcare systems, highlighting the need for context-specific strategies, targeted educational reforms, and policy frameworks aligned with local infrastructure, in accordance with European Union digital health priorities and OECD recommendations for data-driven health systems [43, 44].

Finally, this review contributes to the existing literature by synthesizing knowledge, attitudes, and practices (KAP) among dentists, dental students, and patients, identifying a consistent gap between positive perceptions of AI and its limited clinical implementation. By moving beyond descriptive accounts of AI applications, this work captures behavioral and perceptual dimensions of adoption, offering a more comprehensive understanding of readiness and barriers. Key evidence gaps remain, particularly regarding longitudinal assessments of AI adoption, standardization of KAP measurement tools, and evaluation of educational interventions.

## Limitations

This study has several limitations that should be considered when interpreting the findings. First, the inclusion of studies published only in English and Greek may have resulted in the omission of relevant evidence from other languages, introducing a potential language bias. Second, the substantial heterogeneity in study designs, populations, and outcome measures precluded the performance of a quantitative meta-analysis. Third, the majority of included studies employed cross-sectional designs, which are inherently susceptible to self-reporting bias and limit causal inference. In addition, variability in the definitions and measurement of knowledge, attitudes, and practices (KAP) across studies may affect comparability and synthesis of findings. The search strategy was restricted to selected databases, including PubMed and Google Scholar, complemented by supplementary sources such as the World Health Organization (WHO), the European Commission (EU) and the Organization for Economic Co-operation and Development (OECD), which may have introduced selection bias despite efforts to ensure comprehensive coverage. Furthermore, publication bias cannot be excluded, as studies with statistically or conceptually significant findings are more likely to be published. Finally, a limitation of the search reporting process should be acknowledged, as although the total number of records retrieved was documented, a database-specific breakdown (e.g. PubMed and Google Scholar) was not consistently recorded due to variability in reporting formats and the inclusion of supplementary search sources.

## Conclusions

Available evidence from this scoping review suggests that dentists, dental students and patients demonstrate moderate knowledge and generally positive attitudes toward Artificial Intelligence, while actual clinical use remains limited. The findings highlight a clear gap between awareness and practical implementation, primarily driven by insufficient training, limited hands-on experience, and concerns related to ethics, data privacy, and legal responsibility.

### Future research directions

Future research should focus on longitudinal and interventional studies evaluating how knowledge, attitudes, and practices influence the clinical adoption of Artificial Intelligence in dentistry, as well as the effectiveness of educational and training programs in improving digital competencies and facilitating safe and evidence-based use. In addition, further investigations are needed to establish robust ethical, regulatory, and institutional frameworks, including data privacy, algorithm transparency, and professional guidelines, to support trustworthy integration of AI into clinical practice. Finally, research should explore the impact of AI on clinical decision-making, patient outcomes, and dentist–patient relationships, with particular attention to human-centered approaches, bias mitigation, and the development of clinically representative and validated AI systems.

## Data Availability

All data generated or analyzed during this study are included in this published article No primary datasets were generated.
